# Region-Specific Effects of 10-Hz Transcranial Alternate Current Stimulation Over the Left Posterior Parietal Cortex and Primary Somatosensory Area on Tactile Two-Point Discrimination Threshold

**DOI:** 10.3389/fnins.2021.576526

**Published:** 2021-02-18

**Authors:** Hirotake Yokota, Naofumi Otsuru, Kei Saito, Sho Kojima, Shota Miyaguchi, Yasuto Inukai, Kazuaki Nagasaka, Hideaki Onishi

**Affiliations:** ^1^Institute for Human Movement and Medical Sciences, Niigata University of Health and Welfare, Niigata, Japan; ^2^Department of Physical Therapy, Niigata University of Health and Welfare, Niigata, Japan

**Keywords:** transcranial alternating current stimulation, α-band activity, two-point discrimination, posterior parietal cortex, primary somatosensory cortex

## Abstract

Changes in α-band cortical oscillatory activity (8–13 Hz) affect perception; however, how these changes in the left posterior parietal cortex (PPC) and primary somatosensory cortex (S1), which play different roles in determining the two-point discrimination (TPD) threshold, affect TPD threshold remains unelucidated. Therefore, to determine TPD threshold, we aimed to investigate the function of the left PPC and S1 by applying α-band transcranial alternating current stimulation (α-tACS; 10 Hz). TPD threshold was examined at the pad of the right index finger, contralateral to the stimulation site, in 17 healthy adults using a custom-made, computer-controlled, two-point tactile stimulation device, with random application of either active or sham α-tACS over the left PPC (Experiment 1) and left S1 (Experiment 2). Then, 50% TPD threshold was obtained in the active and sham conditions via logistic regression analysis. Afterward, we compared the difference between the active and sham conditions at 50% TPD threshold in each region and found that α-tACS reduced TPD threshold when applied over the left PPC (*P* = 0.010); however, its effect was insignificant when applied over the left S1 (*P* = 0.74). Moreover, a comparison of the change in 50% TPD threshold among the regions revealed that α-tACS applied over the left PPC significantly reduced TPD threshold compared with that applied over the left S1 (*P* = 0.003). Although we did not reveal the actual changes in cortical activity induced by α-tACS, this is the first empirical evidence that α-tACS applied over the left PPC and left S1 exerts region-specific effects on determining TPD threshold assessed in the contralateral index finger pad by stimulation.

## Introduction

Haptic information from the outside world is input to areas 3b and 1 of the primary somatosensory cortex (S1) by ascending afferent fibers, such as the slowly adapting fibers and rapidly adapting fibers, and is then sent to the secondary somatosensory cortex (S2) and higher-order areas, such as the posterior parietal cortex (PPC) (Inui et al., 2004). Two-point discrimination (TPD), a type of tactile function, has been extensively used in neurophysiological research and clinical practice since it was proposed in 1834 by Weber as a measurement of higher-order perceptual functions. Moreover, TPD threshold increases with aging and certain clinical conditions, such as central or peripheral nerve involvement and non-specific low back pain (Bassetti et al., 1993; Stevens and Cruz, 1996; Heriseanu et al., 2005; Catley et al., 2014a; Harvie et al., 2018). Conversely, it has been reported that TPD threshold decreases by tactile or electrical stimulation to the measurement site (Godde et al., 2000; Ragert et al., 2003, 2008; Tegenthoff et al., 2005; Dinse et al., 2006) and that improvements in TPD positively correlate with the enlarged area of the blood-oxygen-level-dependent signal (Pleger et al., 2003) and gray matter volume in the S1 (Schmidt-Wilcke et al., 2018). Furthermore, the inferior parietal lobule within the left PPC, a major region involved in attentional control (Corbetta et al., 2000; Yantis et al., 2002), is reportedly activated when performing a TPD task rather than during a simple sensory detection task (Sripati et al., 2006; Akatsuka et al., 2007, 2008; Pleger et al., 2016). Therefore, both S1 and PPC play important roles in the TPD process.

Furthermore, alpha (α)-band oscillatory activity (8–13 Hz) in the cortex reportedly affects the process of sensory perception in the brain. Perceptual performance negatively correlates with the power of α-band activity in the visual (Van Dijk et al., 2008; Iemi et al., 2017) and somatosensory (Haegens et al., 2011b; Lange et al., 2012; Baumgarten et al., 2016; Craddock et al., 2017) domains. The underlying mechanisms of these phenomena comprise α-band activity exerting a gating effect that suppresses signal transduction by increasing its power in areas that are not involved in the task at hand (Jensen and Mazaheri, 2010; Foxe and Snyder, 2011; Jensen et al., 2014; Van Diepen et al., 2019). This modulation of α-band activity is considered to be controlled by top–down attention modulation in both the visual (Thut et al., 2006; Gould et al., 2011; Jensen et al., 2014) and the somatosensory (Jones et al., 2010; Zhang and Ding, 2010; Haegens et al., 2011a) domains. Therefore, in the somatosensory cortex, α-band activity is considered to have an inhibitory role.

Conversely, increased α-band activity is reportedly associated with improved performance requiring sustained attention, such as in a working-memory task, where higher-order functions are involved (Makeig and Inlow, 1993; Dockree et al., 2007; Braboszcz and Delorme, 2011). For example, Dockree et al. (2007) reported that a higher α-band power predicts good sustained attention performance and that the oscillatory α-band activity correlated to it is generated in the parietal and occipital lobes. That is, while the α-band activity in the S1 has an inhibitory role, it may have a facilitative role in the PPC, which is involved in attentional control.

However, recent studies have suggested that modulation of α-band activity affects decision confidence and awareness of sensory experiences rather than sensitivity during discrimination tasks in the visual domain (Samaha et al., 2017, 2020; Iemi and Busch, 2018). In addition, as previously mentioned, the role of α-band activity varied among the studies because it plays different roles depending on the requirements during information processing. Although the S1 and PPC are important regions for somatosensory information processing (Inui et al., 2004; Jones et al., 2009; Haegens et al., 2010; Gundlach et al., 2017) and have been proposed as generators of α-band activity (Dockree et al., 2007; Jones et al., 2009; Halgren et al., 2019), the manner in which they affect TPD, a higher-order somatosensory perception, remains unclear. Owing to the hierarchal requirement of information processing in the S1 and PPC, the role of α-band activity might be different depending on the brain regions; therefore, modulation of α-band activity in these regions may have different effects on TPD performance.

Transcranial alternating current stimulation (tACS) has attracted considerable attention as a non-invasive method to modulate cortical oscillatory activity (Helfrich et al., 2014; Vossen et al., 2015). This type of stimulation can only synchronize oscillatory neural activity in the cortical area beneath the stimulus and artificially enhance cortical oscillation in that frequency range when applied over the occipital (Zaehle et al., 2010; Helfrich et al., 2014; Vossen et al., 2015) and somatosensory (Berger et al., 2018) cortices. However, the effects of α-tACS on somatosensory perception have also been reported to remain unchanged (Sliva et al., 2018) or to decrease after stimulation (Gundlach et al., 2017); therefore, there is no unified view yet. Furthermore, a recent study of tACS on the motor system suggested that the reported effect of tACS is not dominated by transcranial stimulation to the brain but by transcutaneous stimulation of the peripheral nerves (Asamoah et al., 2019); studies on rats and cadavers have reported that high-intensity currents of >4 mA are required to directly phase-entrain brain rhythms (Vöröslakos et al., 2018). Because we have not measured actual brain oscillatory activity during α-tACS and have not conducted an electrical field simulation, there were many limitations to our design to strongly address in our initial hypothesis. Although the exact mechanism of tACS is still under investigation, a previous study reported that α-tACS increases somatosensory α-band activity immediately after stimulation (Berger et al., 2018). Further, another study reported that tACS may at least impact circuit dynamics (Sliva et al., 2018); therefore, we conducted the present experiments based on the hypothesis that α-tACS may increase α-band activity.

Considering these findings, although the change in the actual oscillatory brain activity caused by α-tACS remains unknown in the present study, we hypothesized that α-tACS may decrease TPD threshold when applied over the left PPC because increased α-band activities are reported to facilitate sustained attention (Dockree et al., 2007; Clayton et al., 2018). Conversely, α-tACS’ application to the left S1 may increase TPD threshold because it inhibits information processing to the higher-order regions (Jensen and Mazaheri, 2010; Foxe and Snyder, 2011; Jensen et al., 2014; Van Diepen et al., 2019). We tested our hypothesis by measuring TPD threshold, a higher-order somatosensory function that requires sustained attention, after applying 10-Hz α-tACS to the left PPC (Experiment 1) and S1 (Experiment 2), wherein these regions were believed to play different roles in information processing (Corbetta et al., 2000; Yantis et al., 2002; Akatsuka et al., 2007, 2008). Furthermore, by comparing the differences in TPD thresholds in the two regions based on the results of Experiments 1 and 2, we found the cortical region that effectively improved TPD following α-tACS of either the left PPC or S1.

## Materials and Methods

### Participants

Overall, 21 healthy college students (12 men and nine women; age, 20.5 ± 0.8 years) with no history of neurological, orthopedic, or psychiatric disorders and who were not receiving any medication during the experimental period were included in this study. All participants underwent the experiments via the application of α-tACS to the left PPC (Experiment 1) and left S1 (Experiment 2). In the TPD task, four participants who answered two points to a one-point stimulus were excluded from the analysis to maintain the consistency of the task; as a result, final analysis included 17 participants. This experiment was conducted in accordance with the Declaration of Helsinki and was approved by the Ethics Committee of Niigata University of Health and Welfare (18264-19092). Full written informed consent was obtained from all participants.

### tACS

**FIGURE 1:**
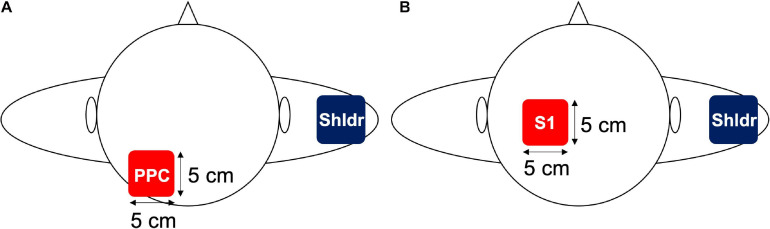
Electrode placement in Experiments 1 and 2. **(A)** In Experiment 1, an electrode was placed over the left PPC, which is located at P3 in the international 10–20 system, whereas the reference electrode was placed over the contralateral shoulder. **(B)** In Experiment 2, an electrode was placed over the left S1, which is located 2 cm posterior to the C3 in the international 10–20 system, whereas the reference electrode was placed over the contralateral shoulder.

Transcranial alternating current stimulation was delivered using a battery-driven, constant current stimulator (Eldith, neuroConn GmbH, Ilmenau, Germany) through a pair of saline-soaked surface sponge electrodes (5 × 5 cm, 25 cm^2^). One of the electrodes was placed over the left PPC (P3 according to the international 10–20 method; Experiment 1) or left S1 (2 cm posterior to C3; Experiment 2), whereas the other electrode was placed on the lateral side of the contralateral shoulder (Figures 1A,B). The reason for placing the reference electrode on the lateral side of the shoulder is that phosphenes reportedly occur during low-frequency tACS at low frequencies, which may affect the results of the measurements (Raco et al., 2014). Specifically, it is possible that the presence of phosphenes may alert the participants about the application of tACS or sham stimulation during the experiment; as a result, the correct answer rate may be affected by the input of sensory information unrelated to the task during the TPD task. Previous studies have solved this issue by placing a reference electrode on the lateral side of the shoulder; therefore, we adopted the same method in this experiment (Mehta et al., 2015; Miyaguchi et al., 2018). Although Mehta et al. (2015) reported that the extracephalic reference resulted in increased stimulation effects of the subcortical brain regions, it has also been reported that the reference electrode placed over the contralateral shoulder to the transcranial direct current stimulation (tDCS) for the dorsolateral prefrontal cortex (DLPFC) improves attentional bias compared with that placed over the left DLPFC (Shahbabaie et al., 2018). Moreover, the current density by tDCS remained the same regardless whether the position of the reference electrode was on the right cheek or right shoulder (Im et al., 2012). Therefore, we assumed that similar results were obtained with tACS and decided to use the extracephalic reference electrode. tACS was applied at 10 Hz with a sinusoidal wave with a constant current intensity of 1.0 mA (peak-to-peak). The impedance was maintained at <10 kΩ during the stimulation in accordance with the latest tACS guidelines (Antal et al., 2017).

### TPD Threshold

**FIGURE 2:**
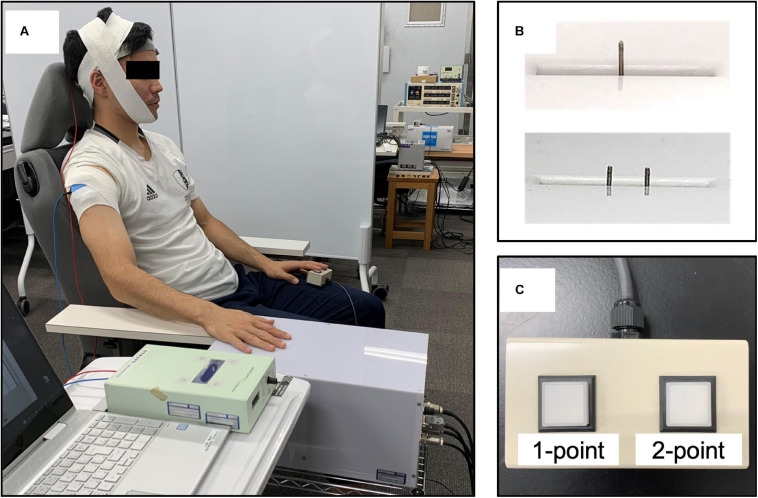
Participants’ position and the two-point tactile stimulation device. **(A)** The participants were seated on a fixed chair with a backrest, with the shoulder and elbow joints slightly flexed and the forearm in a pronated position. **(B)** The stimulus conditions (stimulus speed, 10.0 mm/s; stimulus penetration depth, 1.0 mm; stimulus presentation time, 1.0 s; and stimulus interval, 5 s) were fixed, and pin spacing was randomly assigned (one point, 0 mm and two points, nine different places, ranging from 1.0 to 5.0 mm at 0.5-mm intervals) using a custom-made computer-controlled two-point tactile stimulation device. **(C)** Answer button: participants were asked to respond about whether they perceived two points only when they were confident that there were two points, and an uncertain stimulus or one point was classified as one point.

For TPD measurements, the participants were seated in a resting position on a chair with a backrest and the right shoulder and elbow joints were placed in a slightly flexed position, whereas the forearm was positioned in a pronated position (Figure 2A). A custom-made, two-point tactile stimulator (Takei; Niigata, Japan) that can control stimulus conditions at a given value using a computer was used to randomly present a total of 10 stimuli to the right index finger pad, which is the contralateral side of the stimulated hemisphere, including one point (0 mm between the stimulus pins) or two points (nine stimuli ranging from 1 to 5.0 mm at 0.5-mm intervals; Figure 2B). We explained the instructions to the participants that two points should only be considered if they could be clearly identified as two points, whereas other vague stimuli and one point should be defined as one point. The participants were instructed to respond as quickly as possible when they recognized the stimulus by pushing a button held by the left hand. The stimuli were controlled by a computer under the following conditions: stimulus speed, 10.0 mm/s; stimulus penetration depth, 1.0 mm; and stimulus presentation time, 1.0 s, which were the optimal measurement conditions of TPD, as obtained in our previous study (Yokota et al., 2020) (Figure 2C). Moreover, it was reported that the α-band activity decreased in the hemisphere opposite to the one in which visual or tactile attention was directed; however, in this study, we attempted to increase the α-band activity in the left hemisphere, which perceives information from the right index finger (Van Dijk et al., 2008; Haegens et al., 2010; Jensen and Mazaheri, 2010). Furthermore, TPD threshold reportedly decreased by gazing at the measurement site during the measurement (Kennett et al., 2001; Moseley and Wiech, 2009; Catley et al., 2014b); therefore, we considered that these conditions could affect the measurement results. To minimize these effects, the participants were instructed to relax and look at a fixed point set at the height of each participant’s eyes, at a distance of ∼1.5 m, at all times during the experiment.

### Experimental Procedures

**FIGURE 3:**
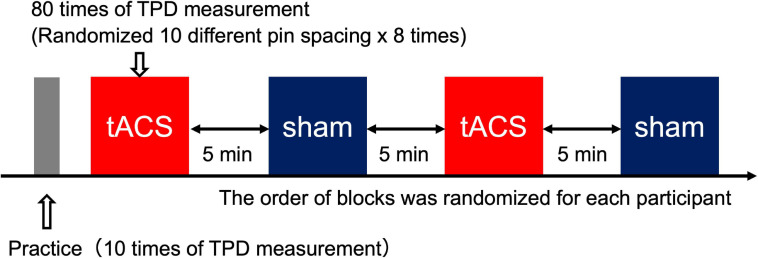
Experimental procedure. Each block included 80 trials (five trials × 18 intervals). There were four blocks in total (two blocks for the tACS condition and two blocks for the sham condition). The order of the blocks was randomized for each participant to minimize order effects. In total, 160 trials (80 trials × two blocks) from each condition were used for subsequent analysis.

All participants first participated in Experiment 1 and then participated in Experiment 2 at least 1 week after the completion of Experiment 1. First, the participants performed 10 practice trials to understand how the stimulus would be presented and how they should respond to the stimulus during the TPD task. After the practice period, TPD was measured in four blocks: two blocks for each of the active and sham conditions, in which 10 different stimulus intervals were presented eight times in each block to obtain 160 measurements for each condition. In the tACS condition of Experiments 1 and 2, tACS was initiated 1 min before TPD measurements, and the TPD task was performed while receiving consistent stimulation during the block and was stopped immediately after the end of TPD measurements (all participants completed TPD measurements within 8 min). In the sham condition, stimulation was initiated 1 min before the onset of the TPD task but was only applied for 20 s (10 s of fade in and 10 s of fade out) to avoid stimulation of the cortex during the TPD task. The order of the blocks was randomly assigned for each participant and a 5-min break was introduced between blocks to avoid any carry-over effects from the previous block (Figure 3).

### Data and Statistical Analyses

To analyze the data, the distance between the pins and the correct answer rate were plotted on the *X*-axis and *Y*-axis, respectively, following which we fitted a binomial logistic regression model [“glmfit” function on MATLAB (MathWorks Inc.) with “binomial” and “logit” settings] to the data to draw a psychometric function. Thresholds at the correct rates of 25, 50, and 75% were calculated for each stimulus condition (tACS or sham) based on the psychophysical curve via logistic regression analysis. Moreover, 50% threshold was defined as TPD threshold and 75% threshold minus 25% threshold was defined as just the noticeable difference (JND), indicating discrimination sensitivity, as previously described (Otsuru et al., 2019). Moreover, the differences in 50% threshold between the tACS and sham conditions were calculated for the PPC and S1 by subtracting the 50% threshold calculated in the tACS condition from that calculated in the sham condition. The findings were compared to identify the cortical region that effectively improved TPD after α-tACS of either the left PPC or left S1.

For statistical analysis, we performed the Shapiro–Wilk test to verify the normal distribution of each stimulus region for 25, 50, and 75% thresholds, followed by the two-tailed paired *t*-test with correspondence between stimulus conditions (tACS or sham). Moreover, we analyzed discrimination sensitivity using statistical software (SPSS; IBM) in both Experiments 1 and 2. Furthermore, the differences in the 50% threshold recorded in the left PPC and left S1 were compared using the two-tailed paired *t*-test. To directly compare the possible regions and to demonstrate the reliability of the TPD measures used in our experiments, we used the two-tailed paired *t*-test to compare the means of the 50% threshold data obtained from the sham conditions in Experiments 1 and 2. The significance level was set at 5% for all statistical analyses. Data are expressed as mean ± standard error (SE).

## Results

### Experiment 1. Effects of tACS Over the PPC on the TPD Threshold

**FIGURE 4:**
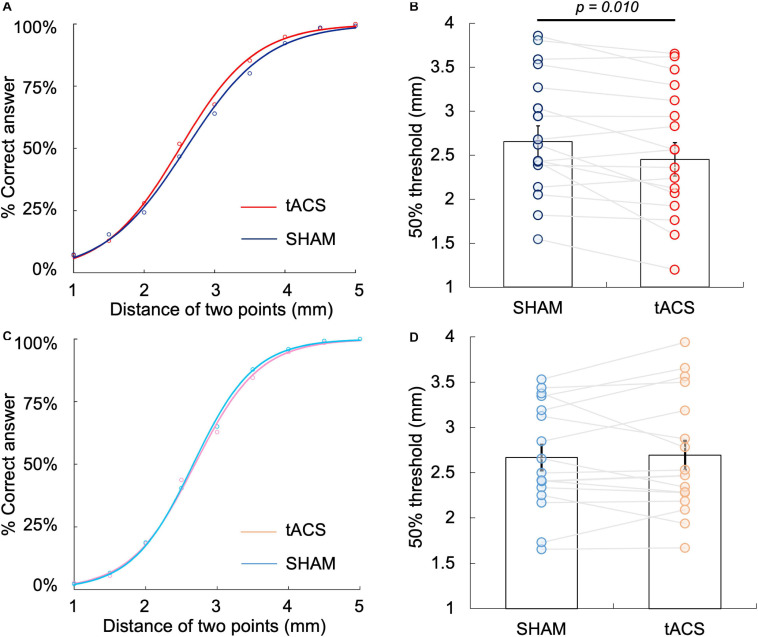
Differences in 50% TPD threshold between the tACS and sham conditions at the left PPC and left S1. **(A)** Psychophysical curve of the tACS and sham conditions at the left PPC in Experiment 1. **(B)** Comparison of 50% TPD threshold between the tACS and sham conditions in Experiment 1, where the *X*-axis indicates TPD threshold and *Y*-axis indicates the percentage of correct answers. The 50% TPD threshold in the tACS condition was significantly lower than that in the sham condition (*P* = 0.010). **(C)** Psychophysical curve of the tACS and sham conditions at the left S1 in Experiment 2. **(D)** Comparison of 50% TPD threshold between the tACS and sham conditions in Experiment 2, where the *X*-axis indicates TPD threshold and the *Y*-axis indicates the percentage of correct answers. There was no significant difference in 50% threshold between the tACS and sham conditions (*P* > 0.05).

Figures 4A,B show the psychophysical curve fitted via logistic regression analysis obtained in Experiment 1 as well as 50% TPD threshold, which was calculated from the curve. The 50% TPD threshold (mean ± SE) was 2.45 ± 0.78 mm in the tACS condition and 2.65 ± 0.74 mm in the sham condition. Moreover, TPD threshold decreased in 13 (76.5%) of the 17 participants during the α-tACS condition compared with that during the sham condition. The two-tailed paired *t*-test between the two groups revealed a significant decrease in the tACS condition compared with the sham condition [*t*_(16)_ = 2.54, *p* = 0.01, *r* = 0.54] (Figures 4A,B). JND was 0.68 ± 0.17 and 0.81 ± 0.25 in the tACS and sham conditions, respectively, with no significant differences between them.

### Experiment 2. Effects of α-tACS Over the S1 on the TPD Threshold

Figures 4C,D show the psychophysical curve fitted via logistic regression analysis obtained in Experiment 2 as well as 50% TPD threshold, which was calculated from the curve. The 50% TPD threshold (mean ± SE) was 2.69 ± 0.66 mm in the tACS condition and 2.67 ± 0.59 mm in the sham condition. Although compared with the sham condition, TPD threshold increased in 11 (64.7%) of the 17 participants during α-tACS application, there was no significant difference between the conditions, according to the two-tailed paired *t*-test [*t*_(16)_ = –0.34, *p* = 0.74, *r* = 0.08] (Figures 4C,D). JND was 0.56 ± 0.21 and 0.58 ± 0.25 in the tACS and sham conditions, respectively, with no significant differences between the two conditions.

### Comparison of the Differences in α-tACS-Induced Threshold Between the Left PPC and Left S1

**FIGURE 5:**
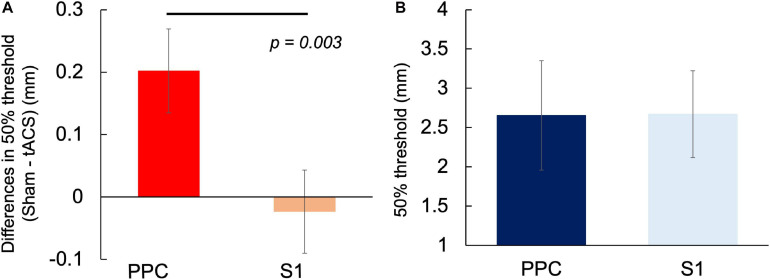
Comparison of the differences in α-tACS-induced threshold change and 50% TPD threshold in the sham condition between the left PPC and left S1. **(A)** Significant differences in threshold differences (50% threshold in the sham condition – 50% threshold in the tACS condition) were observed between the left PPC and the left S1 (*P* = 0.003). **(B)** No difference was observed in 50% threshold between the sham condition (*P* > 0.05).

Based on the results obtained in Experiments 1 and 2, we compared the differences in α-tACS-induced threshold (50% threshold in the sham condition – 50% threshold in the tACS condition) in the left PPC and left S1. The differences in α-tACS-induced threshold in the left PPC and S1 were 0.2 ± 0.28 and –0.02 ± 0.27 mm, respectively, with the changes being significantly larger in the left PPC than in the left S1 [*t*_(16)_ = 3.507, *p* = 0.003, *r* = 0.66, two-tailed] (Figure 5A). However, there was no significant difference in the sham conditions between the regions [*t*_(16)_ = 0.014, *p* = 0.93, *r* = 0.00, two-tailed] (Figure 5B). Therefore, α-tACS over the left PPC effectively lowered the TPD threshold compared with that over the left S1. Moreover, a comparison of the mean of 50% TPD threshold in sham conditions between Experiments 1 and 2 revealed no statistical difference, which helped ameliorate the reliability of the TPD measures used in our experiments.

## Discussion

The present study aimed to clarify the effects of non-invasive brain stimulation over the left PPC (Experiment 1) and left S1 (Experiment 2), which were suggested to be related to TPD, on TPD threshold in the right index finger pad using 10-Hz α-tACS. The results of Experiment 1 illustrated that 10-Hz α-tACS of the left PPC, which is the area that plays a vital role in the TPD process and is responsible for attentional control, decreased TPD threshold. In contrast, in Experiment 2, similar stimulation with α-tACS over the left S1, which is suggested to be involved in tactile signal transfer to the higher-order regions of TPD, yielded no changes in TPD threshold. Furthermore, a comparison of the effects of α-tACS on the left PPC and left S1 based on the differences in 50% TPD threshold revealed that α-tACS applied over the left PPC significantly decreases TPD threshold compared with that applied over the left S1.

A possible explanation for the improvement of TPD following 10-Hz α-tACS over the left PPC is the increased α-band activity caused by α-tACS. Previous studies have shown that α-tACS enhances α-band activity at the stimulated region (Zaehle et al., 2010; Helfrich et al., 2014; Vossen et al., 2015; Berger et al., 2018); therefore, α-band activity at the left PPC might have increased under the tACS condition in the present study; this suggests that this increase in α-band activity improves TPD. This result is consistent with that of previous studies related to the effect of α-band activity on performance during cognitive tasks (Dockree et al., 2007; Braboszcz and Delorme, 2011). Dockree et al. (2007) reported that individuals who performed well on a working-memory task requiring sustained attention for an average of 24 blocks of 4.7 min exhibited a significant increase in α-band activity in the occipitoparietal region during the task. Moreover, in tasks requiring sustained attention for a long period, such as a 28 min × 2 session auditory discrimination task and a 20 min × 3 block respiratory-rate counting task, the α power decreased when participants provided incorrect answers during the task on the simultaneously measured EEG in the same individuals (Makeig and Inlow, 1993; Dockree et al., 2007; Braboszcz and Delorme, 2011). Furthermore, in a temporal order judgment task using electrical stimulation to the left and right index fingers, a 10-Hz α-tACS applied over the PPC, similar to the present study, reportedly improved the discrimination ability, suggesting the involvement of an increased α-band activity in the PPC in the cognitive task (Otsuru et al., 2019). As the TPD task used in this study is a cognitive task requiring prolonged and sustained attention (for approximately 10 min in four blocks, with a 5-min rest period between blocks), the α-tACS-induced increase in α-band activity in the left PPC might have promoted sustained attention to the task and improved task performance. In other words, because α-band activity is considered to exert a gating effect that suppresses signal transduction by increasing its power (Jensen and Mazaheri, 2010; Foxe and Snyder, 2011; Jensen et al., 2014; Van Diepen et al., 2019), increased α-band activity can be deemed to inhibit the left PPC and prevent switching attention away from the task to distractors (Yantis et al., 2002).

Unlike Experiment 1, α-tACS of the left S1 in Experiment 2 did not yield any change in TPD. Similar to visual attention, previous studies have shown that when an electrical stimulus is administered to the index finger, the α-band activity in the S1 on the opposite side of the stimulus decreases (Haegens et al., 2010; Gould et al., 2011). This is important because γ-band activity in the high-frequency region (30–150 Hz), which is important for information transmission, is periodically suppressed by α-band activity in a phase-dependent manner. This is referred to as cross-frequency phase–amplitude coupling (Klimesch et al., 2007; Osipova et al., 2008; Haegens et al., 2011b; Roux et al., 2013; Jensen et al., 2014). Therefore, in the region involved in the task, it is considered that information transfer is facilitated by a burst of γ-band activity that occurs during the trough of α-band activity (Jensen and Mazaheri, 2010; Jensen et al., 2014; Bonnefond and Jensen, 2015; Gips et al., 2016). Moreover, increased α-band activity in the S1 is reportedly correlated with the decreased frequency of neural firing in macaque monkeys and decreased excitability in human MEG and fMRI studies (Osipova et al., 2008; Haegens et al., 2011b; Scheeringa et al., 2011). These data suggest that α-band activity in the S1, which is a relay point of haptic information, needs to decrease physiologically to increase neural firing, thereby facilitating information transfer and improving TPD. In contrast, if the α-band activity in the S1 was decreased during the application of α-tACS, similar to the previously reported decrease in α-band activity after α-tACS application (Gundlach et al., 2017), it may facilitate information transfer and decrease TPD threshold. However, in this study, no significant difference in TPD threshold was observed when α-tACS was applied over the S1. Therefore, our results did not support our initial hypothesis that α-tACS increases α-band activity during stimulation, similar to the previous findings in the visual and parietal cortexes (Helfrich et al., 2014; Vossen et al., 2015; Berger et al., 2018), and thus, disturbed information transfer. That is, the α-tACS over the S1 used in this experiment may not have an effect on α-band activity. However, recent studies have suggested that discrimination sensitivity is not affected even though α-band activity increases in the visual domain (Samaha et al., 2017; Iemi and Busch, 2018; Samaha et al., 2020). Therefore, further studies are warranted to clarify the causal relationship between α-tACS and α-band activity in the S1.

Furthermore, on comparing the differences in TPD threshold because of α-tACS (50% threshold in the sham condition – 50% threshold in the tACS condition) applied over the left PPC and left S1 in Experiments 1 and 2, respectively, the amount of change in the left PPC was significantly larger than that in the left S1. Akatsuka et al. (2008) demonstrated that the inferior parietal lobule in the left PPC selectively showed significantly higher activity during the TPD task in a study that compared cortical activity during the TPD task with the task of discriminating the intensity of stimuli using fMRI. Because increases in α-band activity are critical for cognitive tasks requiring sustained attention (Makeig and Inlow, 1993; Dockree et al., 2007; Braboszcz and Delorme, 2011; Otsuru et al., 2019) and considering that the PPC plays a pivotal role in attentional control (Corbetta et al., 2000; Yantis et al., 2002), our result supports previous results that the left PPC plays an important role in the TPD process (Akatsuka et al., 2008). Moreover, the S1 is important in the process of information processing of TPD and decreased α-band activity must be further decreased to increase neural activity in the S1 such that it can facilitate signal transfer to higher-order regions, such as the PPC. Although this appeared to be paradoxical, the hierarchical mechanism of increases and decreases in α-band activities is controlled by a top–down regulation (Sadaghiani and Kleinschmidt, 2016; Van Diepen et al., 2019); this appears to be crucial for the optimal function of TPD. Therefore, although we did not measure the actual α-band activity during α-tACS application and the focal increment in α-band activity in each cortical region was not supported, it might have been separately modulated by α-tACS to some extent in our experiment, as proposed in previous studies (Zaehle et al., 2010; Helfrich et al., 2014; Vossen et al., 2015; Berger et al., 2018); it might have affected TPD processing, thereby differently shifting TPD threshold depending on the role of α-band activity in each region in the TPD process.

The reliability of the TPD measurement method was a major issue in the present study, as previous studies questioned the reliability of TPD as a measure of perceptual function (Catley et al., 2013; Tong et al., 2013). Slowly adapting I fiber and rapidly adapting I fiber are the primary afferents involved in TPD (Johansson and Vallbo, 1979; Darian-Smith and Kenins, 1980; Craig and Lyle, 2001). Moreover, the frequency of firing of these afferents, including their receptors, changes according to stimulus intensity (Vega-Bermudez and Johnson, 1999a, b). Therefore, the manner in which the stimulus pins are applied during measurements may affect the test results. To overcome this limitation, we used a custom-made two-point tactile stimulator that can control the stimulus speed and stimulus penetration depth of the stimulation pins at the time of measurement to arbitrary values on the computer, thereby providing a comprehensive measurement of TPD threshold. Accordingly, we reported that the threshold value was the lowest at a stimulus speed of 10.0 mm/s and a stimulus penetration depth of 1.0 mm, which represents a reliable measurement method for studying the effects of these interventions (Yokota et al., 2020). There was no significant difference in the mean 50% threshold in the sham conditions between Experiments 1 and 2. This indicates that the TPD measurement method used in this experiment was indeed reliable. Therefore, the TPDs used in this experiment clearly demonstrate the region-specific effects of α-tACS on the left PPC and left S1 because they may accurately reflect the changes in higher-order perceptual functions caused by α-tACS. In the future, the potential clinical applications of these results, such as whether similar results can be obtained in patients with impaired TPD, should be explored.

This study had some limitations in its experimental method. Previous studies have reported changes in cortical oscillatory activity induced by α-tACS (Zaehle et al., 2010; Helfrich et al., 2014; Berger et al., 2018); therefore, we attempted to increase α-band activity in the left PPC and S1 by administering 10-Hz α-tACS. However, we did not record the actual brain oscillatory activity in the cortical region. Therefore, another recently reported mechanism of α-tACS-induced improvement of the TPD, i.e., the entraining of the cortical neurons by the rhythmic activity from the skin’s peripheral nerves under the electrode input via the sensory pathway (Asamoah et al., 2019), should be considered. It is also reported that in rats and cadavers, high-intensity currents exceeding 4 mA are needed to directly phase-entrain brain rhythms (Vöröslakos et al., 2018). In this manner, the entrainment of the cortical activity caused by α-tACS in the present study might not only be focal under the electrode because the rhythmic activity was transmitted via the sensory pathway. Moreover, we have not measured actual brain activity during α-tACS or simulated electrical field distribution; therefore, it is not possible to explain exactly how α-tACS affects cortical oscillatory activity in the left PPC and left S1. Furthermore, the placement of the reference electrode should be considered. Although we placed the reference electrode over the shoulder contralateral to the stimulating electrode to minimize the occurrence of phosphenes, according to previous studies (Mehta et al., 2015; Miyaguchi et al., 2018), the placement of the distant electrode reportedly impacts not only the area under the electrode but also the surrounding area (Faria et al., 2011; Karabanov et al., 2019). The extracephalic reference also increases the stimulation effects on subcortical brain regions (Mehta et al., 2015). Furthermore, the current flow and electrical field distribution by the α-tACS montage used in this experiment were not simulated; therefore, the effects of α-tACS might not be less focal than expected. Therefore, elucidating whether the effects of α-tACS were focal to the left PPC and impossible on the left S1 and that there may be some overlap between the S1 and PPC is essential. However, α-tACS was found to exert different effects over the left PPC and left S1; therefore, the specific reactions of each region could be demonstrated to some extent. Detailed studies, such as those using a device that can simultaneously measure brain oscillatory activity during α-tACS and perform a simulation of the electrical field distribution, are warranted to confirm these results in the future.

## Conclusion

The present study reveals that the use of 10-Hz α-tACS over the left PPC effectively reduces TPD threshold; however, a similar stimulation over the left S1 does not yield any significant changes. The effect of 10-Hz α-tACS on the determination of TPD threshold was region-specific in each cortical region involved in TPD. However, our results are speculative because actual cortical activity was not measured; therefore, additional studies are warranted to confirm the causal relationship between α-tACS in the left PPC and sustained attention or α-tACS in the S1, and information processing during the determination of TPD threshold using a more precise experimental paradigm, such as the concurrent measurement of cortical activity, during 10-Hz α-tACS.

## Data Availability Statement

The raw data supporting the conclusions of this article will be made available by the authors, without undue reservation.

## Ethics Statement

The studies involving human participants were reviewed and approved by the Niigata University of Health and Welfare. The patients/participants provided their written informed consent to participate in this study.

## Author Contributions

HY and HO conceived the study and designed the experiments. KS and SK performed the experiments. NO interpreted the data. SM, YI, and KN helped draft the manuscript. All authors approved the final version of the submitted manuscript.

## Conflict of Interest

The authors declare that the research was conducted in the absence of any commercial or financial relationships that could be construed as a potential conflict of interest.
